# Entomopathogenic Fungi for Pests and Predators Control in Beekeeping

**DOI:** 10.3390/vetsci9020095

**Published:** 2022-02-21

**Authors:** Roberto Bava, Fabio Castagna, Cristian Piras, Vincenzo Musolino, Carmine Lupia, Ernesto Palma, Domenico Britti, Vincenzo Musella

**Affiliations:** 1 Department of Health Sciences, University of Catanzaro Magna Græcia, 88100 Catanzaro, Italy; roberto.bava@unicz.it (R.B.); c.piras@unicz.it (C.P.); studiolupiacarmine@libero.it (C.L.); palma@unicz.it (E.P.); britti@unicz.it (D.B.); musella@unicz.it (V.M.); 2 Interdepartmental Center Veterinary Service for Human and Animal Health, CISVetSUA, University of Catanzaro Magna Græcia, 88100 Catanzaro, Italy; 3 Pharmaceutical Biology Laboratory, Department of Health Sciences, Institute of Research for Food Safety & Health (IRC-FISH), University of Catanzaro Magna Græcia, 88100 Catanzaro, Italy; v.musolino@unicz.it; 4 Etnobotanical Conservatory of Castelluccio Superiore, 85040 Potenza, Italy; 5 Mediterranean Etnobotanical Conservatory Sersale (CZ), 88054 Catanzaro, Italy; 6 Nutramed S.c.a.r.l. Complesso Ninì Barbieri, Roccelletta di Borgia, 88021 Catanzaro, Italy; 7 Department of Health Sciences, Institute of Research for Food Safety & Health (IRC-FISH), University of Catanzaro Magna Græcia, 88100 Catanzaro, Italy

**Keywords:** *Apis mellifera*, *Varroa destructor*, *Aethina tumida*, Vespidae, biological control, entomopathogenic fungi, honey bee welfare and health

## Abstract

The emergence of resistance to chemical drugs in beekeeping is becoming a phenomenon of widespread concern. One promising alternative to the use of chemicals is entomopathogenic organisms that are environmentally friendly and are capable of stopping the expression of resistance once it has evolved. In the recent past, the scientific community has carried out several experiments addressing the use of microbiological control agents. In particular, experimental studies using entomopathogenic fungi have had more success in honey bee research. With their adherence properties and their ability to digest the cuticle and overcome the host defense mechanism, they could be a suitable ingredient in bioacaricides. Several promising fungi have been identified in the search for effective means to control pest populations. The data obtained from the different experiments are interesting and often favorable to their use, but there are also conflicting results. The aim of this review is to describe the state of the art on the topic under investigation.

## 1. Introduction

Honey bees are among the most numerous and efficient pollinating species which, through their activity, are directly or indirectly responsible for a third of our food [1,2]. The breeding of these insects also provides employment opportunities in rural areas and valuable products [3,4,5]. Therefore, their protection is extremely important. Despite their value, the loss of many colonies has occurred in recent years. The underlying causes are not fully known, but can certainly be attributed to more than one factor [6]. Among them are some parasites such as *Varroa destructor*, *Aethina tumida*, *Nosema* spp. that attack the colonies causing severe damage [7,8,9].

Synthetic chemicals are employed to control the populations of these parasites. The choice of these molecules is based on their efficiency and ease of use. However, these pharmacological preparations may contaminate hive products and negatively affect the health of honey bees [10,11]. Furthermore, *Varroa* mites are developing drug resistance, which is reported to be increasing [12,13,14]. All these reasons, combined with a widespread awareness of issues related to environmental protection, the greater attention of consumers to the nature of finished agricultural products, and an ever greater evolution of international legislation in an eco-friendly direction, have favored the development and introduction of management practices and drugs with low environmental impact [15,16].

In recent years, scientific research has focused its attention on the use of entomopathogenic microorganisms, particularly entomopathogenic fungi. Entomopathogens can contribute to reducing selective pressure for pesticide resistance development in pest populations [17,18]. They also have the unquestionable advantage of creating few resistance phenomena [19]; treated mite populations have a lower probability of developing resistance because of the multiple target receptors involved in fungal infections, as contrasted to the use of acaricides.

However, the use of microbiological control agents remains limited to niche contexts. This is correlated with inconsistencies in their performance, the narrow spectrum of action of the bioinsecticides available, their high specificity and the lack of associated technologies that amplify their effect [20]. Laboratory and field studies on entomopathogenic fungi in beekeeping are contradictory.

Laboratory studies have shown, for example, that some *Beauveria* and *Metarhizium* isolates cause significant mortality among honey bees [21,22]. For instance, it has been reported that *B. bassiana* has the potential to infect not only *Varroa* mites, but also bee pupae when applied in laboratory bioassays [23]. When administered in the field, the entomopathogens showed no negative effects on honey bees [24]. Mortality in treated subjects can obscure the potential use as biological control agents. Although hymenopterans may be susceptible, it has nevertheless been shown that social species can avoid infection or minimize its effects [25,26,27]. From another perspective, entomopathogenic fungi have often shown good effectiveness against *Varroa* mites.

It is imperative to evaluate and select entomopathogens with greater selectivity of action for target parasites. Honey bee and their larval forms should be affected little or not at all and, therefore, factors such as special formulations or specific application methods should be implemented to eliminate this possibility. This overview summarizes studies that examine the efficacy of fungal biocontrol agents for *Varroa* spp., *A. tumida* and Vespidae in beekeeping.

## 2. Entomopathogenic Fungi (EPF)

The fungi are a kingdom of eukaryotic heterotrophic organisms. As heterotrophic organisms, they obtain nourishment from the external environment by absorbing products through their walls [28]. In addition to the well-known macroscopic fungi (such as mushrooms and molds), the kingdom includes many microscopic organisms such as yeasts and spores. Fungal cell walls typically are rigid and contain mainly chitin and glucans. Although some species are unicellular (e.g., yeasts), fungi are mainly multicellular and filamentous, with tubular morphology and a septate mycelium or without partitions. They give rise to modular forms with indeterminate growth. Fungal reproduction is complex. It has been estimated that a third of all fungi reproduce using more than one method of propagation. Fungi can reproduce asexually or sexually [29]. The asexual reproduction occurs with the formation of special reproductive cells called spores, as result of mitosis in the parent cell (binary fission, budding and fragmentation); sexual reproduction occurs through the union of sex organs, cells or nuclei forming sexed spores, and the elements involved recombine their genetic information.

In the life cycle of a fungus, the anamorphic state during which the fungus reproduces asexually and the telomorphic state of sexual reproduction can be present, excluded or alternated. The most common way used to assign a fungus to a given group is based on the characteristics of sexual reproduction or using molecular data [30]. Pathogenic insect species are few in the phyla Basidiomycota, Blastocladiomycota, Chytridiomycota and Kickxellomycotina, while a consistent number are recorded in Ascomycota and almost complete dominance occurs in Entomophthoromycota [31].

Many common and important entomopathogenic fungi belong to the order Hypocreales of the Ascomycota [32]. These include the asexual (anamorph) phases *Beauveria*, *Isaria*, *Hirsutella*, *Metarhizium*, and the sexual (teleomorph) state *Cordyceps*. The term entomopathogen refers to any member of the kingdom that can infect insects and other terrestrial arthropods, such as mites, ticks, and spiders. The heterotrophic metabolism forces them to a type of life dependent on a host and, depending on whether the relationship is neutral, of harm or advantage for the host organism, they are divided respectively into saprophytes, parasites, and symbionts. We will mainly deal with parasitic entomopathogen fungi which are harmful to the host.

Numerous fungal microorganisms are pathogenic for many insect and nematode species and are able to control the natural populations of these by limiting their spread [33]. There are more than one thousand species that infect and parasitize insects. Entomopathogenic Hypocreales are opportunistic pathogens highly adapted to infect insects and mites as a result of adaptations developed over time, such as the ability to overcome the host’s immune system defenses and the production of cuticular enzymes and degrading substances [34]. They are globally and widely distributed in nature, ubiquitous across all environmental matrices, and can be easily grown in mass. Because they occur naturally, they can be considered generally environmentally friendly, with low to no residual toxicity to food and constitute a minimal concern for human safety [35,36]. Consequently, they have been developed as microbial insecticides for the control of many important pest arthropods in agriculture, forestry and urban environments in different countries [37].

### 2.1. Infection and Pathogenic Mechanisms

A suitable host becomes infected when it comes into contact with the spores. Properly, infection begins when the propagules adhere to the cuticle of a sensitive host [38]. The adhesion of the spores to the epicuticle of the host, the subsequent germination and formation of the penetration structures (e.g., appressoria) are critical phases of the infection process [38]. The penetration sites appear as dark and melanotic areas of the epicuticle [39]. Entry requires the combined action of enzymatic degradation mechanisms and mechanical pressure. Many entomopathogens rely on the production of hydrophobic conidia to achieve rapid attachment to the waxy epicuticle.

The binding process is also promoted by molecules synthesized by the fungus called adhesins [31]. This initial attack is immediately followed by the secretion of a mucus, with strong adhesive characteristics, and degradation enzymes [40]. Entomopathogenic fungi produce a variety of cuticle-degrading enzymes [41]. Proteases (among which are trypsin, chymotrypsin, esterase, collagenase and chymoelastase) are important for the penetration of *Metarhizium anisopliae* and other fungi. Other enzymes, including endoproteases, esterases, lipases and chitinases, are involved [42]. The action of the various enzymes can be supported by the secretion of organic acids. For example, the Basidiomycota are known to produce oxalic acid, while the genera *Rizophus* and *Aspergillus* produce fumaric, lactic, malic, citric and gluconic acids [43]. Proteases (Pr1 and Pr2) and aminopeptidases are linked to the formation of appressoria [44,45].

**Figure 1:**
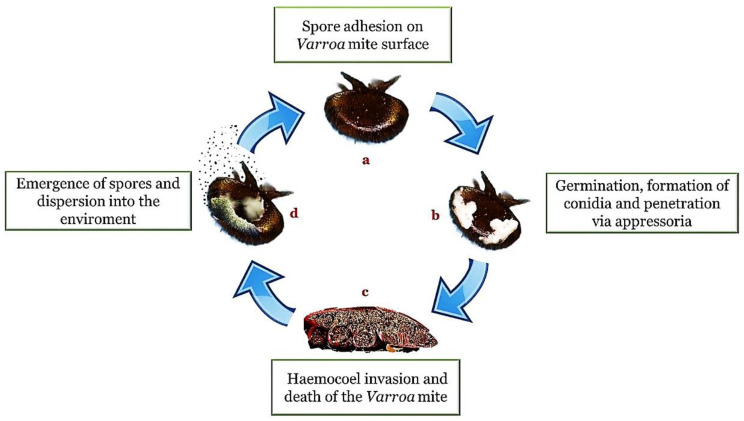
Entomopathogenic infection cycle (method of action). In presence of a suitable substrate and a favorable environment, the adhesion of the spores (**a**) is followed by germination, the formation of hyphae and structures such as the conidia and the appressorium (**b**). Once penetrated inside the body cavity (haemocoel), the fungi cause a depletion of nutrients and a destruction of the tissues until the death of the host (**c**). The cycle is completed with emergence on the surface of the body for the propagation of the infectious elements in the external environment (**d**).

The appressorium is a specialized structure that represents a kind of narrow penetration peg useful for concentrating physical and chemical energy on a very small area of the cuticle [46]. A successful attachment is followed by the growth of fungal hyphae within the cuticle interstices and the subsequent entry into the hemocoel. Within the hemocoel there is a transition from filamentous hyphal growth to the formation of small hyphal bodies with thin walls, similar to yeasts or protoplasts, which circulate in the hemolymph and proliferate. The advantages of this cellular form are the increase in nutrient acquisition rate and the ability to avoid the immune response system. Once the immune system is avoided, septicemia occurs. When the nutrients are depleted, the fungus returns to mycelial growth to invade the host’s internal tissues and organs (Figure 1).

Insect death is the result of several factors, such as mechanical damage, nutrient depletion and the production of toxins in the insect’s body (toxicosis) [47]. Several fungal toxins have been reported to be harmful to insect health. Paralysis, slowness, and reduced responsiveness to external stimuli in fungal-infected insects are symptoms attributable to the action of neuromuscular toxins. Toxins such as beauvericin, beauverolide, bassianolide and isarolide have been isolated from *B. bassiana* [48]. *Metarhizium* spp. has been reported to produce different destruxins, with varying levels of activity and virulence, and cytochalasin [49].

In addition to having an important role as suppressors of the immune response (prevention of nodulation and inhibition of phagocytosis), destruxins depolarize the muscle membrane of lepidopterans and influence the function of insect hemocytes [50]. In general, the role of secondary metabolites is to facilitate the settlement of the pathogen in the host, causing paralysis and interrupting the physiological processes of the host and its immune responses. Due to the infection, the insect body is first damaged and then destroyed by the depletion of nutrients and the development of hyphae within it.

As a consequence of the progressive infection, the initially soft insect body stiffens due to the absorption of liquids by the fungus. The infection process is long and the length of time required for it to occur is approximately 6–14 days [51]. After the death of the host and the exhaustion of nutrients, the hyphae of the fungus emerge from the corpse through the less sclerotic regions: holes of the body and intersegmental membrane. Spores are then produced on the outside of the corpse, partially or completely cover it and allow the conidia to spread and infect other individuals. Conidial dispersion is passive and relies primarily on wind, but other factors, such as rain, can also play a role in diffusion. Inside the hive, infected individuals of the population facilitate the mobilization of spores. After the conidia have been dispersed to another host, the cycle of infection begins again. The death of the insect can occur after a few days, with a variable time correlated to the fungus involved and the number of infecting spores.

### 2.2. Host Defense Mechanisms

Entomopathogens have developed a series of mechanisms aimed at establishing infection but, in a sort of coevolutionary arms race, the hosts have implemented a series of defense mechanisms useful to fight the infection. In addition to the limitations imposed by the environment, the fungal spores must overcome host defense mechanisms. The cuticular barrier and the peritrophic membrane covering the intestine represent two initial lines of defense [52]. Although the epicuticle is an excellent substrate for fungal colonization, some of its constituents have been shown to possess antifungal properties. In particular, some secreted cuticular lipids represent an evolved defense mechanism useful for inhibiting adhesion and germination [53]. Protease inhibitors are also produced at this level, inhibiting the enzymatic activity of the pathogens. During penetration, to reduce hemolymph leakage the hemostatic response is activated. This process involves proteins such as lipophorins, vitellogenin-like proteins, and calcium-dependent trans-glutaminases. The fungal penetration of cuticle is also followed by the activation of the prophenoloxidase cascade in epidermal cells, which will lead to the final synthesis of melanin. Melanin plays an important role in preventing the insect cuticle against fungal invasion. However, melanization is a line of defense against slow and weak growth of the pathogen but is not very effective against the more virulent strains. After a successful penetration into hemolymph, fungi propagate as yeast-like blastospores or hyphal bodies [54]. Following this, the host’s cellular and humoral defense is activated. The cellular immune response relies on circulating hemocytes with the mechanisms of phagocytosis, nodulation, encapsulation, melanization, and production of protease inhibitors. The cellular response mechanism, which hemocyte cells put in place both when the cuticle is perforated by a foreign body and to eliminate targets too large to be phagocytosed (as in the case of parasites, protozoa, and fungi), is encapsulation. The mechanisms of the functioning of the insect immune system have been studied mainly in *Drosophila* and, subsequently, have been applied to other genera [55]. In particular, there are three differentiated hemocyte types: plasmatocytes, which make up most of the cells in circulation and act as phagocytes of bacteria; crystal cells, involved in the melanization reaction and that produce prophenoloxidase; lamellocytes, which mediate the immune encapsulation reaction to large infectious objects that are too large for phagocytes to ingest [56,57].

Massive production of hemocytes in the hematopoietic organs is induced within hours of infestation, resulting in an increase in the number of lamellocytes. These begin to build a series of cell layers around the non-self, forming the hemocyte capsule. They lose their cytoplasmic content, which includes the phenoloxidase that allows melanization to start. A key enzyme in melanin synthesis is phenoloxidase (PO), synthesized in the form of zymogen (prophenyloxydase or PPO) by different types of hemolymph cells, such as crystal cells and lamellocytes [58]. The recognition of pathogens and/or lesions leads to the activation of a series of serine proteases, with consequent proteolytic cleavage of the inactive PPO and formation of the active PO, which catalyzes the formation of phenolic and quinone compounds [59].

Once formed, melanin is deposited around invading microorganisms [59]. Inside the capsule, the non-self is killed by the production of cytotoxic radicals, such as reactive oxygen species (ROS) and reactive nitrogen species (RNS), or by asphyxiation [60,61]. Quinonic substances and ROS are produced as intermediates during the synthesis of melanin and have a cytotoxic action. The invasion of the hemocoel triggers the humoral immune response system, which leads to the production of a series of host antifungal immune effectors. The systemic immune response takes longer to establish than the cellular response [62]. It represents the last line of immune defense against persistent infections [63].

Antimicrobial peptides (AMPs) are the main effectors of the humoral immune response. AMPs are peptides, generally cationic, which in the hemolymph can reach very high concentrations [64]. The main production site of these molecules is the fat body [65]. Several types of antimicrobial peptides have been identified including defensins, cecoprins, diptericins, apidaecins and hymenoptaecins. The defensins have a broad spectrum of action: they have cytotoxic activity against Gram-positive and some Gram-negative bacteria, fungi and protists [66]. The trigger of the immune response is in fact activated by the recognition of the intruder by receptors such as Pattern Recognition Receptors (or PRRs) and ꞵ-glucan-binding proteins, which recognize molecular motifs associated with pathogens (Pathogen Associated Molecular Patterns), such as lipolysaccharides (LPS), peptidoglycans (PGN) and beta-1.3 glycans [67]. The humoral immune response against a fungal invasion is mediated by the Toll pathway.

Recognition of fungal microorganisms by the Toll-like receptor (TLR) triggers a cascade reaction which, involving signaling intermediates, leads to the translocation of transcription factors from the cytoplasm to the nucleus. This process results in the production of antimicrobial peptides (AMP) in the fat body and their subsequent secretion within the hemolymph. The Toll pathway has receptors capable of recognizing PGNs that have L-lysine residues [68].

The other two major pathways activated by fungal infection are the JNK and the JAK/STAT pathways, both of which are involved in the synthesis of antibacterial AMP and stress/injury response proteins. Other important immune-related molecules produced by insects are lysozime, apolipophorin III, hemocyanin and transferrin. The expression of transferrin is increased during *B. bassiana* and *M. anisopliae* infection suggesting its important role in the infection.

In addition to cellular and humoral immunity, social insects show a higher level of defense which finds its expression in grooming behavior. The grooming behavior and behavioral changes in infected insects, such as behavioral fever exhibited by infected grasshoppers [69], may help the host against fungal colonization and the development of the disease. Another example is the hygienic behavior exhibited by the honey bee in the presence of *Ascophaera apis* infection: the workers identify larvae infected and remove them. From another point of view, the grooming behavior may be harmful. The infectious propagules removed with mouthparts are stored in the infrabuccal cavity within the buccal chamber and infection can occur if inocula germinate. However, it has been hypothesized that chitinase secretions within the oral cavity have a fungistatic activity.

Although not yet fully understood, entomopathogenic fungi have developed evolutionary strategies and adaptations that allow them to evade or overcome their host’s antifungal cell defense system. Among them is the assumption of a morphological change once inside the hemocoel. For Hypocreales, this involves the transition from hyphal growth to the formation of small thin-walled hyphal bodies, similar to the hyphal bodies of yeast (also known as blastospores). This form speeds up the dispersion through the hemolymph and avoids the detection by pathogen recognition molecules, although they are still susceptible to plasmatocyte phagocytosis [70]. More virulent fungal strains are also capable of overcoming the hemocyte response causing a reduction in the number of granulocytes [71,72]. Many of the entomopathogenic fungi in the Entomophthoromycota phylum take the form of protoplasts (similar to blastospores), which helps them evade detection by hemocytes [73].

Furthermore, entomopathogenic fungi mask the immunogenic carbohydrates of the surface to avoid immune stimulation and secrete secondary metabolites that cause paralysis, interference with the physiological processes of the host and, mainly, suppression of the immune responses.

## 3. Detection and Preparation of Entomopathogenic Fungi for Experimental Assays

Entomopathogenic fungi can be isolated directly from different matrices (such as hosts, soil, mulch, and plants) using selective media or insect bait methods. Thorough sample preparation is extremely important for the isolation of endophytic fungi. In nature, entomopathogenic Hypocreales can be harvested directly from the corpses of insects on which the fungus has sporulated. In general, environmental conditions of humidity and temperature prevent the fungus from growing on the host cuticle and producing spores. If no external sporulation has taken place, dead hosts may be placed in a suitable environment to enable fungi to produce hyphae or conidia outside the host cuticle. A first wash with surfactants is followed by disinfection of the host surface with sterilizing agents such as sodium hypochlorite (NaClO) and/or 70–95% ethanol [74].

Hosts are then placed in Petri dishes and exposed to high relative humidity at room temperature to allow fungal growth in the cuticle. If the identity of the fungus is known, it is recommended to use a selective medium for that particular fungus. Otherwise, any medium used for propagation can be used, such as sabouraud dextrose agar with yeast extract. Furthermore, antibacterial agents such as tetracycline, chloramphenicol or streptomycin are frequently used for bacterial inhibition. Crystal violet is commonly added to inhibit the growth of Gram-positive bacteria. However, it is the inhibition of contaminant fungi that is more problematic than bacteria. Fungi in the genera *Trichoderma* and *Rhizopus* grow rapidly and can obscure colonies of Hypocreales. Selection in fungal growth can be achieved by modifying the media with dodine, cyclohexamide or benomyl [75]. Incubation should take place in darkness at ambient temperature for 5–7 days. *B. bassiana* is easily cultured on solid and liquid media such as Saubouraud Maltose Agar Yeast Extract medium (SMAY), the Potato Dextrose Agar (PDA), and the Dextrose Potato Broth (PDB); *M. anisopliae* on solid medium such as Sabouraud’s dextrose agar with 1% yeast extract (SDAYE) and liquid media such as yeast extract/dextrose broth.

The in vitro assessment of the biocontrol potential of a fungal strain against a possible host involves initial decontamination of the target in 1% sodium hypochlorite solution, physiological saline or distilled water with 0.1% Tween 80. In many cases the insect is infected with fungal pathogens by immersing them in a spore/conidial suspension for few seconds [76,77,78]. Subsequently, infected hosts are dried on sterile gauze and then placed on Petri dishes at room temperature and relative humidity >80%. In other experiments, *Varroa* mite spp. were forced to walk on gauze soaked in a suspension of conidia or blastospores, then placed on Petri dishes and incubated in the usual conditions of temperature and relative humidity [79,80]. Infected mites are provided with honey bee larvae for their sustenance and nutrition.

## 4. Laboratory Assay and Field Studies for *Varroa destructor*, *Aethina tumida* and Vespidae Control

### 4.1. Use of Fungal Entomopathogens in the Control of Varroa destructor Infestation

As early as 1992, Evans suggested that mites are excellent hosts for fungal pathogens: the soft body and humid microclimates in which they usually live facilitate colonization and fungal growth [81]. Much of the initial research on an appropriate fungal species and strain that may have a high virulence against *Varroa destructor* has focused on fungal pathogens of other insects/arthropods [82,83]. Therefore, none of the pathogens initially tested were isolated directly from *V. destructor*. The rationale behind this decision was that, since many of these entomopathogenic fungi have a wide range of hosts, some of them could potentially be pathogenic to *Varroa*. Furthermore, the registration of fungi products is often expensive and time-consuming, and difficulties can arise from the direct isolation of entomopathogens from hosts found dead in nature.

*B. bassiana*, for example, can easily grow on dying or just dead insects. The relief of its presence on the dead mite does not necessarily imply that the fungus in question killed the mite. The mites with the vital conidia on the cuticle may have died for other reasons and, in the days preceding the collection, the conidia may have developed on the corpse, generating confusion about the cause of the death. Sterilization of the exterior surfaces of the body may eliminate this possibility, but not necessarily. On the other hand, the superficial sterilization of mites with damaged bodies, but still dead from mycosis, could mask the real cause of death.

Among the entomopathogenic fungi, the most common pathogens of mites present in nature belong to phylum Entomophthorales [84]. Other common fungal pathogens belong to the genera *Hirsutella* and *Metarhizium*, both of which are frequently tested for microbial control of ticks [85,86,87]. In general, experimental studies have shown the susceptibility of *Varroa* mites parasite to fungal pathogens [23,83,88,89,90]. Shaw et al. [90] hypothesized that this could be due to the evolution of these mites in a relatively pathogen-free environment such as that of the hive.

The first experiments against *V. destructor* were carried out with different strains of fungi belonging to the genera *Hirsutella* and *Metharizium* [83]. Most of these studies used aqueous conidia suspensions (mostly conidia suspended in water plus Tween 80) conveyed by means of strips or dust formulations [91,92,93,94,95]. In laboratory conditions, the conidial suspension dispersed in the aqueous medium usually had a concentration of 10^7^ conidia/mL. When entomopathogenic fungi were applied in field studies, a higher dose was used to effectively control mites. After treatment, the initial phenomena of mortality in the mites occurred in three or more days. On average, *V. destructor* peak mortality occurred mostly 5–7 days after conidia were applied [23,83], and Kanga et al. [83] reported that the mites were still infected 42 days after treatment. The results obtained in these first studies were promising, inasmuch the mortality of the mites was highly correlated with mycosis [90,91].

In laboratory tests, through electron microscopic observation of the deceased infected *V. destructor*, Peng et al. [79] established that the membranous arolium of the mite leg sucker is the focal point of the fungal infection. The spores therefore appeared not to adhere easily to the *Varroa* mite cuticle, except for the legs. However, it should be pointed out that in the experiment the fungal infection was induced by forcing the mites to walk for 5 min on the fungal culture. [79].

The potential acaricidal activity of the *Metharizium* was also investigated by James et al. [92,93] in two field trials. The research team sprayed frames in colonies with an aqueous suspension of spores. Although, some infected mites were recovered, the spores survived very shortly and the tests were considered ineffective [92]. The first efforts to isolate and verify the biocontrol activity of entomopathogenic fungi isolated directly from *Varroa* mites were made by Meikle et al. [23], who isolated strains of *B. bassiana* from the hive environment [96].

Previously, scientific research had not considered B. *bassiana* as a good candidate for the biological control of *Varroa* mite. *B. bassiana* is known to be susceptible to high temperatures, which inhibit germination and fungal growth [97,98,99]. Meikle et al. [95] also determined the effects of the application of B. *bassiana* conidia, formulated in two variants, namely with wax powder/conidia and flour/conidia, on the weight of the colony, on the mass of adult bees, on the covered brood and on honey, and on the fall of *Varroa* on sticky boards. They observed a greater fall in infected mites in both treatments than in control groups [100]. The research group then verified the effectiveness of two subsequent applications of *B. bassiana*. No impact on colony health was observed after two successive applications of the formulation containing *B. bassiana* conidia and an increase of the mite’s fall was registered [96]. The two administrations made it possible to better cover the entire brood cycle, allowing the fungi to infect even newly emerged honey bees [101].

However, multiple applications would increase the effectiveness but also the costs, making biological products less attractive for mite control. Other reports on the natural occurrence of the entomopathogenic fungi *B. bassiana* on *Varroa* mites were published by García-Fernández et al. [80] and Steenberg et al. [102]. In particular, *B. bassiana* fungi were isolated from *Varroa* mites picked up directly from capped worker brood cells [102]. In the same years, Rodriguez et al. [89] were selecting in the laboratory fungal strains that could best adapt to the microenvironment present inside the hive, measuring the radial growth of different colonies in contact with nutrient medium at temperatures of 30 and 35 °C. At the highest temperature tested, most isolates were inhibited from growing. Three B. *bassiana* and M. *anisopliae* isolates were the exception. These isolates were selected, and, in a laboratory study, the pathogenicity was evaluated on V. *destructor* by applying a suspension of 10^7^ conidia/mL The most effective isolate was M. *anisopliae* Qu-M845 [89]. This particular strain has been tested in field trials [103]. Treatments were performed using three application methods: a) conidia stamped on filter paper located on frames inside the hive; b) conidia sprinkled between the frames; and c) conidia dispenser path located at the entrance of the hives. The second method of application had the best results [103].

In subsequent years, the entomopathogenic fungus *Clonostachys rosae* was tested in a laboratory test with unsatisfactory results [78]. Afterwards, Sinia and Guzman-Novoa [104] tested the acaricidal efficacy of entomopathogenic fungi in combination with thymol. Mortality of mites was close to 82%, but this value was not significantly different from thymol treatment alone used as a control [104]. Lastly, two valuable studies carried out in more recent years are worthy of mention. The first aimed at evaluating the effectiveness of secondary metabolites of fungal origin [105], while the second aimed at evaluating the possibility of applying direct evolution to obtain more resistant and performing fungal strains [106].

In particular, the efficacy of destruxins, secondary metabolites of fungi, was verified by Lodesani et al. [105] through tests to evaluate the toxicity of crude and purified destruxin (DTX), fractions A, B, CE and D, towards *V. destructor* and *A. mellifera*. Mortality of mites treated with crude DTX and DTX B and CE was higher than control mites in all performed trials. Enthusiasm for promising laboratory results on mites has been tempered by toxicity tests on honey bees; at higher concentrations, the experiments resulted in a significantly higher mortality rate compared to the control groups, suggesting the need for further research [105]. However, after lowering the dose, *Varroa* mortality remained unchanged while honey bee mortality declined. These results show that secondary metabolites are elements that could be taken into account in addressing *V. destructor* infestation. Extraction of destruxins is quick and inexpensive. Once extracted, they remain stable for a month when stored at temperatures between 4 and 8 °C. It should also be noted that the action of destruxins is not affected by the microenvironmental characteristics of the hive and that their use would eliminate the possible toxicity of other components excluded in the extraction process. Therefore, further research is required to investigate fractions, doses, solvents and methods of administration, which may contribute to the control of *V. destructor* populations without harming honey bees [105].

With regards to direct evolution, a strain of *Metarhizium brunneum* was subjected to repeated rounds of selection using both direct evolution in laboratory incubators and repetitive selection in colonies of full-size honey bees. These operations allowed create of a strain more resistant to the microenvironmental conditions present inside the hive and therefore more persistent in carrying out its infectious function against *Varroa* [106]. In any case, none of the above-mentioned in vivo studies resulted in a significant difference in the density of phoretic mites between the treated hives and the control groups. Positive exceptions to this rule are the work of Kanga et al. [91,101], in which colonies were treated in the absence of brood and this may have contributed to the success of the treatment, and the treatments in combination with thymol performed by Sinia and Guzman-Novoa [104]. In the latter case, the overall effectiveness is due to the synergy of the action.

### 4.2. Susceptibility of Honey Bee to Entomopathogenic Fungi

No less important are the possible effects on adult honey bees and the larval forms. Laboratory studies involving direct inoculation of spores into the body of adult bees and brood have shown a high mortality of treated subjects compared to untreated control groups [22,23,78,90,107]. Unlike these observations in laboratory studies, in the tests carried out in the field, no adverse effects of the biopesticide treatment were recorded towards adult honey bees, the immature stages and, in general, in relation to the growth rate of the colony [91,95,96,100,108,109,110]. This is very likely to be traced back to several causes: (1) the hygienic behaviors implemented by social insects within a colony; (2) the protective activity of compounds such as propolis that help bees to extinguish the infection and in detoxification [111,112,113]; (3) the method of administration which often involved spraying or blowing the spores on the honeycombs—in an open system with uncontrolled conditions, such as the environment [95]. These examples show the limitations of in vitro studies and underscore the need to implement field studies in addition to in vitro studies. With reference to the sublethal effects, the study carried out by Cappa et al. [114] highlighted how bees exposed to *B. bassiana* have altered cuticular hydrocarbons which make it difficult for nestmates to recognize them. In this sense, the recent studies conducted by Araya et al. [115] and Omuse et al. [116] add other pieces to the knowledge acquired over the years. They investigated the behavioral responses and the eventual mortality of pollinating insects following the use of entomopathogenic fungi [115,116]. In the experiments conducted by Araya [115], *B. bassiana* (Balsam) *Vuillemin* and *M. anisopliae* (Metschnikoff) were applied to nurse bees, kept in an environment at 30 °C and in dark conditions, at a concentration of 10^8^ conidia/mL, a concentration previously known for its pathogenicity on *V. destructor*. The behavioral responses of the subjects were recorded. Both fungi did not affect several normal parameters of honey bee behavior in the laboratory, including duration of walking periods, rest, feeding, drinking, communication with antennae (touching other honey bees) and grooming [115]. Omuse et al. have compared the mortality that occurs following exposure to entomopathogens in the honey bee and in *Meliponula ferruginea* (Hymenoptera: Apidae). In particular, isolates from strains belonging to *Metarhizium* and *Beauveria* were tested. Under the same experimental conditions, the conidial acquisition by *A. mellifera* was greater than by *M. ferruginea*. Furthermore, no isolates had significant effects on *M. ferruginea*, while three isolates caused mycosis in *A. mellifera* with mortality rates close to 18%. These results show that the hymenopterans have a different susceptibility to the fungal species and to the strains used. In Table 1, laboratory and field tests of entomopathogenic fungi against *V. destructor* are reported.

### 4.3. Use of Fungal Entomopathogens in the Control of Aethina tumida Infestation

Many beetles of the Nititulidae family are known vectors of disease-causing pathogens [117]. *Aethina tumida* is responsible for the fermentation of honey in honeycombs and could therefore be a vector of fungal microorganisms. Scavenger lifestyles are common among nititulids that can feed on carrion, rotten fruits, flowers, etc. The evidence that adults and larvae of *A. tumida* feed on fruit and reproduce easily on old and moldy combs suggests that the small hive beetle is able to tolerate various pathogenic microorganisms naturally present in its environment [118]. Despite this, Lundie [119] highlighted, during a laboratory breeding program, a high mortality of adult beetles attributable to an unidentified fungal pathogen [119]. Ellis et al. [118] registered a 32% of mortality in *A. tumida* pupae after contact of mature larvae with pupae killed by a possible pathogen [118,120]. Subsequently, five fungal species were isolated from pupae killed by the aforementioned potential pathogen: Two of these were *Aspergillus niger* and *A. flavus* [121]. These species are commonly isolated from the soil and may infect the pupal stage of *A. tumida* when the larvae leave the hive and burrow to pupate [122,123].

The main concern regarding the use of *Aspergillus* species in the hive is that both species are responsible for fungal diseases in honey bees: stonebrood is a fungal disease caused by *A. fumigatus, A. flavus* and *A. niger*. In addition, fungi from this genus produce toxins that are known to be carcinogenic. In 2006, Mürrle et al. [124] have reported promising results regarding the effects of several species of entomopathogenic fungi on *A. tumida.* In particular, the pathogenicity of a fungal pathogen, closely related to *M. anisopliae* strain FI-203, was first observed during a mass breeding program of beetles. The susceptibility of adult *A. tumida* to three further isolates of other entomopathogenic autochthonous fungi (*B. bassiana*, *H. illustris* and *M. anisopliae*) of South Africa was also assessed. The study demonstrated a considerable susceptibility of *A. tumida* to *B. bassiana* and low pathogenic effects of the other species tested [124]. Leemon and McMahon [125] showed that several *Metarhizium* and *Beauveria* isolates were effective in controlling *A. tumida* larvae and adults in laboratory assays. In particular, *Metarhizium* and *Beauveria* isolates killed more than 70% of larvae within one week and 99–100% of adult beetles within two weeks, respectively [125]. The lack of pathogenicity of *M. anisopliae* and *B. bassiana* towards mammals and humans makes their use promising as possible biological control agents for this beetle. In Table 2, laboratory and field tests of entomopathogenic fungi against *A. tumida* are reported.

### 4.4. Use of Fungal Entomopathogens in the Control of Vespidae

Research into the use of entomopathogenic fungi against other honey bee enemies is limited. The greater wax moth larva, *Galleria mellonella,* is often used as a model insect in various studies on the efficacy of biocontrol agents and some papers have been published on entomopathogenic fungi [126,127,128]. Although discussion of the studies of *G. mellonella* is not the aim of this review, among the latest we can mention the publication of Fergani and Yehia [127]. In this publication, *G. mellonella* has been shown to have a linear percentage mortality when inoculated with progressive serial concentrations of a sporal solution of *B. bassiana,* strain Y-F_ITS1. The mortality was proportional to spore concentration and time exposure.

More interesting for our discussion are the studies on the control of Vespidae by entomopathogenic fungi. The efficacy against vespids, in fact, should make us reflect on the possible risks of introducing these biocontrol agents in beekeeping not only towards bees but also against other Hymenoptera useful to the ecosystem.

For Vespidae, the control and reduction of their impact in Europe is mainly based on the destruction of the nests. The capture of the founding queens is instead a method used in spring aimed at reducing the reactivated subjects that leave the winter shelters to found new colonies. The use of traps and toxic baits is also frequent. Referring to the usage of biological control methods, it should be emphasized that no control strategy has yet become effective. Despite this, it is also useful in this case to take stock of the situation. Among the first studies conducted is that of Harris et al. [129] which tested the biocontrol potential of numerous entomopathogenic fungi isolated from wasp cadavers, nest material and other unrelated insects against *Vespa vulgaris*. The resulting experimental evidence demonstrates the susceptibility of workers and larvae to high concentrations of spores [129]. Since this study just described, publications on this form of contrast have become increasingly rare. An impulse to continue on this path was given by the discovery in Europe of the Asian hornet, *V. velutina* [130]. In 2006, Merino et al. [131] studied the pathogenicity of 29 isolates of *M. anisopliae* and 30 of *B. bassiana*, on workers and males of *Vespula germanica*. Evaluation was performed by administering doses of 1 × 10^8^ conidia/mL of each isolate, in sugary liquid bait. Mortality and sporulation of the isolates of *B. bassiana* Qu-B941 and Qu-B933 were significantly higher, reaching percentages of 90% and 97%, respectively, for Qu-B941 and Qu-B933, with the highest concentration of inoculum [131]. Three years later, Brownbridge et al. [132] examined the fungi *M. anisopliae* and *B. bassiana*, mixed into non-toxic protein bait. They reported the presence of infected larvae in the nest and reduced rates of trafficking to the nest, but these pathogen baits did not have the same fast-acting effect as toxic baits [132]. In a recent study conducted by Poidatz et al. [133], the high virulence of entomopathogenic fungi of the genus *M. anisopliae* and *B. bassiana* against *Vespa velutina* was shown [133]. The same research group has published the first report regarding a *B. bassiana* strain naturally occurring on *V. velutina* [134]. These results bode well for a possible use of entomopathogenic fungi against these insects. In Table 3, laboratory and field tests of entomopathogenic fungi against Vespidae are reported.

## 5. Biotic and Abiotic Factors Affecting the Activity of Fungi

Although the results of the laboratory studies were quite similar and satisfactory, the same cannot be said of the field studies that revealed conflicting data [82,83,90,110]. The effective use of entomopathogenic fungi as a biological control agent is influenced by the environmental conditions in which they are exposed. Their pathogenic power depends on biotic and abiotic factors [88]. Biotic factors include the fungal strain, its physiology, host defense mechanisms, host development stage and cuticular characteristics at the time of application. Abiotic factors include temperature and humidity that influence spore germination and host colonization [75].

Fluctuating temperature ranges affect the germination, growth and yield of the strain used. Optimum temperatures for spore germination and colony growth for most entomopathogenic fungi vary from 20 °C to 25 °C, with host infection and pathogenesis occurring between 15 °C and 30 °C. Temperatures greater than 30 °C therefore inhibit fungal growth and affect pathogenesis. Honeybees finely regulate the temperature inside the colony, generally keeping values between 31 °C and 36 °C. The highest values are obviously recorded at the brood level, where temperature fluctuations are detrimental to the good development of the larval forms [135,136,137]. The temperature in the honey bee colony also changes significantly from summer to winter. In winter, honey bees form a winter cluster and stay active in order to maintain an appropriate temperature between 24 °C and 34 °C [138]. These changes in temperature and changing seasonal conditions must be taken into account in the application of entomopathogenic products under field conditions. Although the conidia of some fungal strains have been shown to germinate and grow at brood temperature, these conditions can still reduce their survival [101]. Thus, the detection of the survival of biological control agents in the microclimate of the hive is a fundamental step [88,136,137]. Isolates with a large temperature range for germinating and growth should be tested. Few isolates of *M. anisopliae* and *B. bassiana* have the potential to tolerate 35 °C [89]. Ouedraogo et al. [139] also found that *M. anisopliae* has a wider growth range and a higher optimum temperature (30 °C) than *B. bassiana* [139].

Almost all published studies on the thermal biology of entomopathogenic fungi are carried out under constant temperature conditions [140,141,142,143], with few studies investigating how fungal activity changes with temperature variations [144]. The response of fungi to temperature change is an inclined bell-shaped curve with a rapid decrease in activity when temperatures rise above the optimal level. A similar growth curve implies that even small changes in temperature can have an enormous impact on infectious capacity. This non-linear temperature response can be modeled, but little has been done to predict changes in EPF performance at fluctuating field temperatures.

In the Davidson et al. [88] experiment, the proliferation of fungi was evaluated at three different temperatures (20, 30 and 35 °C) for 41 entomopathogenic isolates. All isolates grew at temperatures of 20 °C and 30 °C, but only 11 grew at 35 °C. The best fungal growth occurred between 22.9 °C and 31.2 °C. Only three isolates developed well beyond 30 °C. The thermal needs of the isolates examined, potentially pathogenic for *Varroa*, were in line with the temperatures present in the hive in the areas where there is no brood; a limited number of isolates seemed able to develop in areas with male brood [145]. The humidity must also be considered. High humidity is required, and low ambient humidity has been observed to be associated with germination failures in field trials. The application will give more positive results in the presence of high ambient humidity.

Other abiotic factors affecting the infection process include wind and sunlight. Spore dispersal is aided by wind in the natural environment. Sunlight and ultra-violet (UV) radiation can reduce the infectious process. However, these factors have little importance in the conditions of the hive.

Population density and its behavior are more important. The movements and grooming behavior can influence the spread of the pathogen, as demonstrated by the spread of *M. anisopliae* conidia among individual termites by grooming [146]. On the other hand, grooming behavior may promote infection even in conspecifics within the hive in case of low specificity of action of the strain tested.

## 6. Formulations and Methods of Application

The end-use products on the market are physically present as powders or emulsified oils. Within them are often added additives to protect the spores against UV rays, improve the ability to adhere to the foliage, or increase the humidity around the spores. The photoactive dye, phloxine B, has also been suggested to protect against phototoxicity [147]. Moisture absorbers such as calcium chloride, silica gel, magnesium sulphate, white carbon or sodium sulphate are recommended for addition to powdered preparations. Burges [148] recommended flour that acts as a nutritional additive for the germination of fungal conidia and also the use of oil as a hygroscopic and lipophilic material for dry environments [148]. In liquid and powder formulations, oils are added to increase the shelf life of the products and the effectiveness in dry environments. The addition of oils also promotes a better adhesion of spores to the waxy surface of the cuticle. Mineral and vegetable oils are employed. Common and frequently used vegetable oils are corn and cottonseed oil [149].

It is also well known that the method of administration of the drug influences its pharmacological efficacy. In the field tests against *Varroa,* the materials used as carriers for fungi were flour and wax powder. The spray method was the most frequently chosen technique for application. As seen above, this method has the indisputable advantage of not causing damage to adult bees and the brood. The disadvantage is the scarce practicality of use in the field for large-scale treatments of a large number of hives. Soil treatment of fungal spores is the most viable application strategy for *A. tumida*. In the soil, humidity is sufficient to promote conidial germination and mycelial growth allowing the inoculation of fungi to be sustained over time; the conidia in the soil are also protected from harmful solar radiation. The soil also helps to moderate large fluctuations of temperature. However, the antagonistic effect of parasites and soil microorganisms (fungi, bacteria, arthropods) cannot be excluded. Metabolites secreted by microbes can impair the ability of entomopathogenic fungi to infect their host. Investigations of ecological interactions are necessary to develop effective control strategies.

## 7. Conclusions and Outlook

Parasites, particularly *V. destructor* and predators, represent a serious threat to the health, welfare and productivity of *A. mellifera* worldwide [150,151] and they are the main causes of the significant hive losses for beekeeping globally [152]. Currently, the control of parasites is mainly based on the use of chemical drugs [153]. However, parasiticide drug resistance has become a major and growing problem in bee farms in many countries of the world, in particular in USA, Canada, South America, Asia and in some European countries [13,154]. The maintenance of treatment efficacy is very important to ensure high quality of beehive products, honey bee welfare and health in the beekeeping farms. In addition, other important aspects that should direct research efforts towards alternative control strategies are represented by the toxicity of pesticides for the environment, for microfauna and mammals [155,156].

These problems have not been noted with the use of entomopathogenic fungi and studies aimed at evaluating the possible negative effects related to the application of EPF in the environment have not shown negative effects on ecosystems and mammals [157,158,159].

Therefore, the use of entomopathogenic fungi could represent a promising tool to achieve an eco-friendly approach and could be a prospective approach for the near future in beekeeping [160].

The entomopathogenic fungi could be included in the paraphernalia available to the beekeeper, alongside other products with low environmental impact and little tendency to the development of drug resistance, such as organic acids, essential oils and ethnobotanical remedies [161,162,163,164].

However, there are certain limits on the use of bioinsecticides. Primarily, the inoculum has a short shelf life also influenced by external environmental conditions. Moreover, they take 2–3 weeks to kill the target and they need application coinciding with high relative humidity and/or coformulants which reduce the impact of environmental conditions on vitality of spores [148]. In this context, it is necessary to develop new techniques that help to use these microorganisms in a better way. As highlighted for secondary metabolites (destruxins) [105], modern molecular biology techniques can enhance desired traits and increase the efficacy in the field of formulations used. [162]. To increase virulence and resistance to environmental conditions, an interesting solution could be represented by the genetic manipulation of fungi with stronger fungal strains through protoplast fusion and anastomosis techniques [163]. Even nanotechnological formulations of fungal propagules can improve the biological activity of the preparations. The protection and resistance provided by carrier materials and acquired through the microencapsulation process could protect the fungi from adverse environmental conditions when applied in field experiments [164]. Furthermore, in view of the use of entomopathogenic fungi in integrated pest management program, the use of these fungi in association with natural active ingredients could enhance their efficacy [104]. This could pave the way for the use of Green Veterinary Pharmacology (GVP) as an alternative and sustainable method to reduce the use of chemicals and counter the growing phenomena of resistance to one or more pesticides.

In conclusion, the entomopathogenic fungi are important as they are environmentally friendly, they do not cause resistance phenomena in parasites, they are safe for the host and interrupt the life cycle of the parasite [160]. This also reduces the transmission of various pathogens, such as bacteria and viruses, with indisputable benefits for bee welfare and health.

## Figures and Tables

**Table 1:** Synoptic table of laboratory and field tests cited for *Varroa destructor*.

EPF Species	Formulation Type	Application Method	Reference
*Beauveria bassiana*, *Hirsutella* spp., *Metarhizium* spp., *Paecilomyces* spp., *Tolypocladium* spp., *Verticillium lecanii*	Spore solution	Immersion for a few seconds	Shaw et al. [90]
*Hirsutella thompsonii,* *Metarhizium anisopliae*	Liquid	Sprayed frames	Kanga et al., 2002 [83]
*Hirsutella thompsonii* (laboratory test)	Fungal culture	Walk for a few minutes	Peng et al., 2002 [79]
*Metarhizium anisopliae* *Metarhizium anisopliae*	Powder Liquid	Dusted between frames/strips between frames Sprayed frames	Kanga et al., 2003 [91] James et al., 2006 [110]
*Metarhizium anisopliae* *Lecanicillium lecanii*	Liquid	Sprayed frames	Gerritsen% Cornelissen [82]
*Beauveria bassiana*	Powder	Blown between frames	Meikle et al., 2007 [100]
*Beauveria bassiana*	Powder/Flour/carnauba wax powder	Blown between frames	Meikle et al., 2007 [95]
*Lecanicillium lecanii, Beauveria bassiana,* *Metarhizium anisopliae, Hirsutella kirchneri,* *Hirsutella nodulosa* (laboratory test)	Fungal culture	Walk for a few minutes	Fernandez et al., 2008 [80]
*Metarhizium anisopliae*	Powder/Liquid	Filter paper between frames/sprinkled between frames/dispenser path hive entrance	Rodriguez et al., 2009 [103]
*Beauveria bassiana* *and Metarhizium anisopliae*	Powder Dispenser tray		Sinia & Guzman-Novoa, 2018 [104]
*Metarhizium anisopliae/Beauveria bassiana/* *Clonostachys rosea* (laboratory test)	Spore solution	Immersion for a few seconds	Hamiduzzaman et al., 2012 [78]
*Metarhiziumanisopliae, Beauvaria bassiana*	Commercial preparation suspended in water	Sprayed frames	Abdelaal & Hany, 2013 [108]
Destruxins (laboratory test)	Crude and purified destruxins		Lodesani et al., 2017 [105]
*Metarhizium brunneum*	120 g of colonized grain bearing 2.63 × 10^8^ spores per gram.		Han et al., 2021 [106]

**Table 2:** Entomopathogenic fungi (EPFs) active versus *A. tumida*.

EPFs	References
*Beauveria bassiana* *Metarhizium anisopliae*	Muerrle et al. [124] Leemon & McMahon [125]
*Aspergillus niger,* *Aspergillus flavus*	Richards et al. [121]
*Hirsutella illustris*	Muerrle et al. [124]

**Table 3:** Entomopathogenic fungi (EPFs) active versus Vespidae.

Species	EPFs	References
*Vespa velutina*	*Beauveria bassiana* *Metarhizium anisopliae*	Poidatz et al. [133]
*Vespula vulgaris*	*Aspergillus flavus,* *Beauveria bassiana* *Metarhizium anisopliae*	Harris and Harcourt [129] Brownbridge et al. [132]
*Vespula germanica*	*Metarhizium anisopliae* *Beauveria bassiana*	Merino et al. [131]
